# Adult health burden and costs in California during 2013 associated with prior adverse childhood experiences

**DOI:** 10.1371/journal.pone.0228019

**Published:** 2020-01-28

**Authors:** Ted R. Miller, Geetha M. Waehrer, Debora L. Oh, Sukhdip Purewal Boparai, Sheila Ohlsson Walker, Sara Silverio Marques, Nadine Burke Harris

**Affiliations:** 1 Pacific Institute for Research and Evaluation, Calverton, Maryland, United States of America; 2 School of Public Health, Curtin University, Perth, Australia; 3 University of California, San Francisco, California, United States of America; 4 Human Impact Partners, Oakland, California, United States of America; 5 Children’s National Medical Center, George Washington University, Washington, DC, United States of America; 6 Center for Youth Wellness, San Francisco, California, United States of America; University of Bern, SWITZERLAND

## Abstract

**Objectives:**

To estimate the adult health burden and costs in California during 2013 associated with adults’ prior Adverse Childhood Experiences (ACEs).

**Methods:**

We analyzed five ACEs-linked conditions (asthma, arthritis, COPD, depression, and cardiovascular disease) and three health risk factors (lifetime smoking, heavy drinking, and obesity). We estimated ACEs-associated fractions of disease risk for people aged 18+ for these conditions by ACEs exposure using inputs from a companion study of California Behavioral Risk Factor Surveillance System data for 2008–2009, 2011, and 2013. We combined these estimates with published estimates of personal healthcare spending and Disability-Adjusted-Life-Years (DALYs) in the United States by condition during 2013. DALYs captured both the years of healthy life lost to disability and the years of life lost to deaths during 2013. We applied a published estimate of cost per DALY.

**Results:**

Among adults in California, 61% reported ACEs. Those ACEs were associated with $10.5 billion in excess personal healthcare spending during 2013, and 434,000 DALYs valued at approximately $102 billion dollars. During 2013, the estimated health burden per exposed adult included $589 in personal healthcare expenses and 0.0224 DALYs valued at $5,769.

**Conclusions:**

Estimates of the costs of childhood adversity are far greater than previously understood and provide a fiscal rationale for prevention efforts.

## Introduction

A growing body of research demonstrates that Adverse Childhood Experiences (ACEs) affect neurological, immune, endocrine, and genetic regulatory systems, resulting in negative short- and long-term effects on physical and mental health[1–7]. This study’s purpose is to estimate the adult health risk and costs in California during 2013 associated with adults’ prior ACEs. The estimates highlight the importance of ACEs as a health problem and permit cost-outcome analyses of efforts to prevent or treat the toxic stress associated with childhood adversity. Specifically, this paper combines published prevalence-based estimates of annual healthcare costs and disability-adjusted life-years (DALYs) for ACEs-related diseases with estimates of excess risk of disease associated with ACEs based on prior research.

Previous cost studies of childhood adversity have focused on child maltreatment including physical abuse, sexual abuse, psychological abuse, and neglect. Fang et al.[8] estimate the national lifetime economic burden from new cases of fatal and nonfatal child maltreatment to be approximately $124 billion. Bonomi et al.[9] estimate that the long-term health care costs were 21% higher among women with a history of physical or sexual child abuse compared to those without such a history. Brown et al.[10] review the literature on child maltreatment and report estimates of annual healthcare costs in adulthood ranging from $0 to $800 dollars. Comparable estimates are not available for exposure to ACEs which include a broader array of childhood stressors including growing up in a household with domestic violence or with family members with depression. However, Bellis et al.[11] report that adults with a history of ACEs have significantly higher odds of frequent primary, emergency, and inpatient care relative to those with zero exposure.

## Methods

### Data

We estimate healthcare costs of exposure to ACEs in California, by combining prevalence data from four years of the California Behavioral Risk Factor Survey (BRFS) with estimates of healthcare costs during 2013 from Dieleman et al.[12] for those chronic conditions identified as plausibly linked to ACEs in Waehrer et al.[13] The California BRFS is part of the Behavioral Risk Factor Surveillance System (BRFSS), the US system of cross-sectional state-based telephone surveys of residents’ health-related behaviors. State health departments queried residents aged 18 and older in all 50 states and 3 US territories about demographics, risk behaviors, chronic health conditions, and use of preventive services using a standardized questionnaire developed in collaboration with the Centers for Disease Control (CDC). Additional topical modules developed by the CDC are administered in some years by some states, probing topics such as arthritis, depression, and ACEs, with state-developed questions of local interest also allowed. This study is based on 2008, 2009, 2011, and 2013 California BRFS data on ACEs from the California Department of Public Health (CDPH)[14].

### Identifying ACEs-related diseases

Seminal research using data from a California health plan has associated ACEs with increased odds of adverse health outcomes including heart disease, liver disease, and lung disease, with a graded association between the number of ACEs and risk of some outcomes[15–19]. Subsequent studies found support for these results using data from several states as well as global data[20–23]. At the same time, these studies varied in the demographic controls used, and how extensively the ACEs-disease relationship was mediated by health risk factors was unclear. A companion study by our research team used parallel logit models with a common set of socioeconomic controls to examine whether the direct ACEs-health relationship for seven health outcomes persisted net of health risk factors such as smoking, heavy drinking, and obesity[13, 24]. We applied the models to BRFSS data from California and 13 other states. That study also estimated the extent to which ACEs association with health outcomes ran indirectly through smoking, heavy drinking, and obesity by comparing the results from models with and without the three health risk factors. Health outcomes were deemed to be ACEs-related if, (1) net of the three health risk factors, they had a graded, dose-response relationship with the number of ACEs, (2) they were significantly associated with exposure to 4 or more ACEs, and (3) these relationships were consistently observed across the different BRFSS states[13, 24]. Among the seven health outcomes analyzed, five—asthma, arthritis, depression, COPD, and cardiovascular disease–were plausibly linked to ACEs under the criteria listed above. Two other conditions, diabetes and cancer, did not meet our criteria and therefore were excluded from this cost study.

### Estimating excess disease risk associated with ACEs

To estimate the excess risk of disease associated with ACEs exposure, we combined results from logit models in Waehrer et al.[13] with California BRFS data on (1) the prevalence of ACEs-related health conditions for different ACEs exposures and (2) the proportion of CA population exposed to ACEs. Specifically, Table 1 presents the average change in the probability of the health condition for those with ACEs relative to those with zero ACEs using estimates from Waehrer et al.[13]. S1 Table presents the full set of estimates and confidence intervals from that study. To be consistent with the healthcare cost estimates from Dieleman et al.[12] (explained below), we used estimates in columns (1) to (3) of S1 Table, which present the increased probability of disease associated with ACEs exposure from models that do not control for risk factors such as lifetime smoking, heavy drinking, or obesity.

**Table 1:** Fraction of health conditions associated with ACEs in California.

# ACEs	N	ACEs Prevalence ^a^	Outcome Prevalence ^a^	Change in outcome probability ^b^	Excess risk among exposed (%)	ACEs-Associated Fraction in Pop (%)
**Health Risk Factors**						
**Heavy Drinking (≥18 years)**						
0	8,851	39.0%	11.60%			
1	5,030	21.7%	16.30%	2.9	17.79	3.86
2–3	5,191	23.5%	18.80%	4.8	25.53	6.00
4+	3,306	15.8%	23.70%	8.7	36.71	5.80
ACEs Exposure						15.66
**Lifetime Smoking (≥18 years)**						
0	8,874	39.0%	28.20%			
1	5,048	21.6%	36.00%	7.60	21.11	4.56
2–3	5,217	23.5%	39.50%	12.00	30.38	7.14
4+	3,322	15.9%	47.90%	21.60	45.09	7.17
ACEs Exposure						18.87
**Obesity (≥18 years)**						
0	8,679	39.0%	21.10%			
1	4,939	21.6%	23.60%	0.9	3.81	0.82
2–3	5,125	23.6%	26.30%	2.7	10.27	2.42
4+	3,262	15.8%	31.00%	7.1	22.90	3.62
ACEs Exposure						6.87
**Health Conditions**						
**Arthritis (≥45 years)**						
0	5149	42%	32.50%			
1	2848	22%	34.70%	4.9	14.12	3.09
2–3	2828	22%	37.00%	7.2	19.46	4.34
4+	1623	14%	41.60%	13.7	32.93	4.54
Any ACE						11.98
**Asthma (≥18 years)**						
0	8,875	39.0%	11.60%			
1	5,052	21.7%	13.50%	1.7	12.59	2.73
2–3	5,217	23.5%	15.60%	3.6	23.08	5.42
4+	3,322	15.8%	21.60%	8.7	40.28	6.36
Any ACE						14.52
**COPD45 (≥45 years)**						
0	3615	39.6%	3.70%			
1	2151	22.8%	6.20%	2.6	41.94	9.56
2–3	2110	23.2%	9.50%	5.3	55.79	12.94
4+	1237	14.4%	10.50%	6.5	61.90	8.91
ACEs Exposure						31.42
**Cardiovascular Disease (≥45 years)**						
0	6,737	39.0%	10.60%			
1	3,632	21.7%	11.70%	1.9	16.24	3.52
2–3	3,515	23.5%	12.20%	3.3	27.05	6.36
4+	2,053	15.8%	12.70%	5.3	41.73	6.59
ACEs Exposure						16.47
**Depression (≥18 years)**						
0	6,826	37.5%	6.30%			
1	4,032	21.8%	11.40%	4.9	42.98	9.37
2–3	4,178	24.2%	16.60%	10.1	60.84	14.72
4+	2,698	16.5%	27.30%	19.4	71.06	11.73
ACEs Exposure						35.82`

^a^ Weighted estimates from California BRFS data from 2008, 2009, 2011, 2013.

^b^ Measured in percentage points. From columns (1)-(3) in S1 Table, based on Waehrer et al. (2018)

For each disease and category of ACEs (n = 1, 2–3, 4+), we calculated the excess risk of disease due to n ACEs (hereafter referred to as ACEs-Associated Fraction or AAF) as the percentage of the adult population with n ACEs times the predicted change in probability of the problem for adults with n ACEs versus 0 ACEs divided by the prevalence of the problem among adults with n ACEs.

#### Calculating costs

Healthcare spending associated with ACEs during 2013: We applied the ACEs-associated fraction (AAF) of disease for different ACEs exposures to prevalence-based estimates of personal healthcare spending in California by disease during 2013 using national expenditure data from Dieleman et al.[12] for the five plausibly ACEs-linked diseases and the three health risk factors. That study used nationally representative data on patient health system encounters for 140 diseases included in the 2013 Global Burden of Disease (GBD) study[25] and 15 additional conditions that were associated with significant spending but were not underlying conditions of health burden. Four risk factors including obesity and tobacco use were included in this latter category. Dieleman et al.[12] allocated spending for treatment of these conditions to these risk factors while treatment for diseases caused by these conditions was allocated to the diseases themselves. Dieleman et al.[12] reported the share of spending by age category. We used the reported share for those 20 years and older to estimate spending for those aged 18 years and older in our study. We inflated Dieleman’s estimates to 2017 dollars using a price index for personal health expenditures[26]. We estimated costs for California by multiplying the national estimates by 0.114, the CA share of the US personal healthcare expenses in national health expenditure data for 2013[27, 28]. Finally, to calculate adult healthcare costs during 2013 per person aged 18 years and older in California who previously experienced ACEs, we summed costs over the ACEs-linked conditions and divided by the estimated number of persons aged 18 and older in California who were exposed to ACEs (76% of the 38.3 million CA population in 2013 times 61% exposed according to BRFS, where 76% is the proportion of the CA population aged 18 and older in 2013[28].

Health burden associated with ACEs during 2013: We also tabulated the estimated health burden in California during 2013 that was associated with ACEs, with burden measured in DALYs. The 2016 Global Burden of Disease (GBD) study[25, 29] provided prevalence-based DALYs estimates by disease for the US. It defined DALYs as the sum of the years of healthy life lost to the disease during the year plus the years of life lost to deaths from the disease that occurred during the year. We used the study’s California DALYs estimates for 2013 for those aged 20 years and older. Multiplying the DALYs estimates for California by the AAF yielded an estimate of the health burden associated with ACEs, measured in DALYs. As is widely done in cost of illness studies and regulatory impact analyses, we also monetized DALYs in order to present health burden in the same metric as healthcare expenses. To calculate the dollar equivalent, we applied the widely cited estimate of the value of a DALY of $235,855 (in 2017 dollars) used in injury and illness cost estimates[30–33]. Similar to healthcare expenses, we also calculated DALYs and their dollar equivalent per person exposed to ACEs.

#### Share of ACEs-related costs deriving from risk factors

To calculate the share of ACEs-related disease risk that runs indirectly through ACEs associations with health risk factors, we compared the change in probability of health outcomes estimated from logit models that excluded and included all three risk factors—lifetime smoking, heavy drinking, and obesity (see S1 Table). Specifically, from S1 Table, we calculated the percentage difference between columns (1) and (4); (2) and (5); and (3) and (6) for exposure to 1, 2–3, and 4+ ACEs respectively. For example, we calculated the share of 4+ ACEs-related CVD due to health risk factors as 21% (5.35–4.24 divided by 5.35). We applied this factor to the estimate of ACEs-related disease costs to estimate the share due to health risk factors.

## Results

Table 1 presents the fraction of health conditions associated with ACEs. Exposure to ACEs was associated with 35.8% more depression cases in California, followed by 31.4% more COPD cases, 16.5% more cardiovascular disease cases, 14.5% more asthma cases, and 12% more arthritis cases. Among health risk factors, ACEs exposure accounted for almost 19.0% more cases of lifetime smoking in California, 15.7% more heavy drinking, and 7.0% more obesity.

S2 Table presents estimates of personal healthcare spending for California during 2013. Cardiovascular disease had the highest spending during 2013 with $26.9 billion in spending among those aged 18 and older, more than 3 times as high as depression ($7.8 billion), followed by COPD ($6.1 billion), arthritis ($5.9 billion), and asthma ($2.8 billion).

Multiplying the costs in S2 Table times the ACEs-associated fractions in Table 2, exposure to ACEs was associated with $10.5 billion dollars in personal healthcare spending in California during 2013 (in 2017 dollars). Despite the higher ACEs-associated fraction for depression and COPD, ACEs-associated healthcare spending was the highest for cardiovascular disease ($4.4 billion) reflecting the high cost of treating this condition. This was followed by depression ($2.8 billion), COPD ($1.9 billion), arthritis ($708 million), and asthma ($401 million). Among health risk factors, treatment for heavy drinking had the highest ACEs-associated healthcare spending of $169 million. ACEs were associated with almost $40 million in spending for obesity treatment and $2 million for smoking treatment.

**Table 2:** Healthcare expenses and DALYs associated with ACEs exposure in California.

Condition # of ACEs	ACES-Associated Fraction^a^ (%)	Healthcare Spending Associated with ACEs ($)	Health Burden Associated with ACES (DALYs)
**Health Risk Factors**			
**Heavy Drinking**			
1	3.86	41,605,749	
2–3	6.00	64,659,817	
4+	5.80	62,504,490	
ACEs Exposure	15.66	168,770,056	
**Lifetime Smoking**			
1	4.56	517,126	
2–3	7.14	809,624	
4+	7.17	813,105	
ACEs Exposure	18.87	2,139,855	
**Obesity**			
1	0.82	4,753,194	
2–3	2.42	13,980,454	
4+	3.62	20,881,178	
ACEs Exposure	6.87	39,614,826	
**Health Conditions**			
**Arthritis**			
1	3.09	182,938,456	2,370
2–3	4.34	256,702,397	3,325
4+	4.54	268,844,163	3,482
Any ACEs	11.98	708,485,016	9,177
**Asthma**			
1	2.73	75,509,330	1,657
2–3	5.42	149,855,089	3,289
4+	6.36	175,852,408	3,859
Any ACEs	14.52	401,216,827	8,805
**COPD**			
1	9.56	584,562,925	34,028
2–3	12.94	791,325,227	46,064
4+	8.91	545,006,035	31,725
ACEs Exposure	31.42	1,920,894,187	111,817
**CVD**			
1	3.52	949,585,816	46,973
2–3	6.36	1,712,887,016	84,731
4+	6.59	1,776,789,510	87,892
ACEs Exposure	16.47	4,439,262,343	219,595
**Depression**			
1	9.37	728,850,087	22,214
2–3	14.72	1,145,299,680	34,907
4+	11.73	912,039,220	27,798
ACEs Exposure	35.82	2,786,188,987	84,919
**All Outcomes**		**10,466,572,097**	**434,313**

^a^ ACES-Associated Fractions taken from last column of Table 1.

As Table 2 shows, ACEs were associated with approximately 434,000 DALYs in California during 2013. ACEs-related DALYs were highest for cardiovascular disease (almost 220,000 years), followed by COPD (almost 112,000 years), depression (85,000 years), arthritis (over 9,000 years), and asthma (over 8,800 years). With DALYs valued at $235,855, the estimated ACEs-associated DALY losses in California during 2013 were valued at $102 billion dollars (in 2017 dollars).

Table 3 reports the per-capita cost in California during 2013 of ACEs-associated adult health conditions. Overall, 61% of Californians aged 18 and older were exposed to ACEs resulting in $589 of personal healthcare expenses and $5,769 in health burden per exposed adult during 2013. Per-person costs increased with ACEs exposure; the costs per capita for those with 4 or more ACEs are twice as high as costs for those exposed to 1 ACE.

**Table 3:** Per-capita cost during 2013 of adult health outcomes in California associated with prior ACEs exposure.

All Outcomes	CA Exposure	# Adults Exposed to ACEs	Total Health $	Health $/ Person^a^	Total DALYs (years)	Total DALYs ($)	DALYs/ Person ($) ^a^
ACEs Exposure	61.0%	17,755,880	10,466,572,097	589	434,313	102,434,954,231	5,769
1 ACE	21.7%	6,316,436	2,568,322,683	407	107,242	25,293,500,980	4,004
2–3 ACEs	23.5%	6,840,380	4,135,519,304	550	172,315	40,641,425,048	5,336
4+ ACEs	15.8%	4,599,064	3,762,730,110	818	154,756	36,500,028,203	7,936

^a^ Per-capita costs using 2013 population estimate of 38,300,000 for California (Census, 2013) and a 76% share for population 18 years and older.

Table 4 presents the share of ACEs-associated costs during 2013 that run through smoking, heavy drinking, and obesity. For those exposed to 4+ ACEs, these health risk factors account for 25% of the increased probability for COPD, 21% of the increased probability for cardiovascular disease, 17% of the increased probability for arthritis, 11% for asthma, and 7% for depression. Applying these risk factor shares to the ACEs-associated healthcare spending in California reveals that 18.3% (approximately $810 million) of ACEs-related CVD healthcare expenses during 2013 derived from ACEs associations with health risk factors. By contrast, only 4.6% of ACEs-related healthcare spending for depression derived from health risk factors. Overall, $1.6 billion of healthcare spending during 2013 and almost 65,000 DALYs in CA were linked to ACEs-associated health risk factors, equivalent to over 15% of the total health disease burden associated with ACEs.

**Table 4:** Share of ACEs-related health costs and health burden due to risk factors.

# ACEs	% ACEs Association due to Risk Factors ^a^	Healthcare Spending Associated w Risk Factors ($2017)	DALYs Associated w Risk Factors (years)
**Health Risk Factors**			
**Heavy Drinking**			
1	100%	41,605,749	
2–3	100%	64,659,817	
4+	100%	62,504,490	
ACEs Exposure		168,770,056	
**Lifetime Smoking**			
1	100%	517,126	
2–3	100%	809,624	
4+	100%	813,105	
ACEs Exposure		2,139,855	
**Obesity**			
1	100%	4,753,194	
2–3	100%	13,980,454	
4+	100%	20,881,178	
ACEs Exposure		39,614,826	
**Health Conditions**			
**Arthritis**			
1	11.41%	20,873,278	270
2–3	20.51%	52,649,662	682
4+	17.58%	47,262,804	612
Any ACEs		120,785,743	1,565
**Asthma**			
1	9.30%	7,022,368	154
2–3	9.85%	14,760,726	324
4+	11.14%	19,589,958	430
Any ACEs		41,373,052	908
**COPD**			
1	12.38%	72,368,890	4,213
2–3	13.56%	107,303,701	6,246
4+	25.03%	136,415,011	7,941
ACEs Exposure		316,087,602	18,400
**CVD**			
1	18.03%	171,210,323	8,469
2–3	15.67%	268,409,395	13,277
4+	20.87%	370,815,971	18,343
ACEs Exposure		810,435,689	40,089
**Depression**			
1	3.43%	24,999,558	762
2–3	3.39%	38,825,659	1,183
4+	6.95%	63,386,726	1,932
ACEs Exposure		127,211,943	3,877
**All Outcomes**		**1,626,418,766**	**64,839**

^a^ % difference between columns (1) and (4); (2) and (5); and (3) and (6) in S1 Table for 1 ACE, 2–3 ACEs and 4+ ACEs respectively.

### Costs attributable to 4+ ACEs

Thus far, we focused broadly on the excess costs attributable to exposure to ACEs. However, 4+ ACEs have been particularly implicated in epidemiological studies of chronic disease. Our estimates show that exposure to 4+ ACEs was associated with $3.8 billion in healthcare expenses in California during 2013 (Table 3), an estimated 36% of total ACEs-attributable healthcare spending, and approximately 155,000 DALYs.

## Discussion

A growing body of research shows that ACEs can lead to changes in the structure and function of children’s developing brains resulting in chronic dysregulation of the stress response as well as other hormones that regulate functions such as heart rate, blood pressure, metabolism, appetite, and reproduction[34–37], and long-term changes in the brain and immune system leading to increased measures of inflammation and impairments of certain immune functions[38, 39]. Consistent with such physiological changes, studies of health outcomes have shown that ACEs can be plausibly linked to higher odds of asthma, arthritis, cardiovascular disease, COPD, and health risks such as obesity, smoking, and heavy drinking [e.g., 13, 18, 23]. This study provides the first US estimate of the economic burden of these negative health outcomes of ACEs. We estimated that ACEs in California were associated with $10.5 billion in excess personal healthcare spending during 2013, and the loss of 434,000 healthy life years valued at an estimated $102 billion dollars. Although our estimates focused on costs during 2013, costs should be similar during other years. Our estimates illustrate the size of the problem and provide a compelling fiscal rationale for prevention and mitigation efforts directed at the problem of ACEs in California.

The adult health burden from exposure to ACEs during childhood rises with the number of ACEs experienced. In a resource-constrained world, the temptation is to focus ACEs response strongly on those with four or more ACEs. Our results call that strategy into question. The harm gradient is not steep. Although the 26% of ACE-exposed California adults reporting exposure to four or more ACEs incurred a disproportionately high 36% share of the health burden, most of the health burden resulted from exposure to fewer ACEs. The 36% of ACE-exposed adults exposed to only one ACE accounted for 24% of the health burden. Substantially reducing the burden requires a broad net of prevention and treatment.

Prevention of ACEs-attributable illness requires a strategy focused on screening for ACEs and treatment of their effects through collaboration among medical, behavioral health, and public health experts. Primary care health professionals can integrate universal screening of childhood adversity in their clinics, refer patients to mental health or multi-disciplinary treatment, and closely monitor and treat the development of behaviors and illnesses associated with ACEs. Upstream efforts at screening for ACEs should begin in childhood as an approach to reduce exposure and continue into adult primary care to assure that disease prevention and management activities are cognizant of early life trauma. Numerous studies have explored the effectiveness of screening of childhood adversities in primary care, and have seen promising findings overall in improved outcomes related to healthcare costs, quality of care, feasibility, and reduced maltreatment prevalence[40–43]. However, careful consideration of clinical integration of screening for early life adversity is also important and requires ongoing research[44–46]. In addition to screening, health care providers should address behavioral risks associated with ACEs, such as alcohol and drug use, smoking, weight problems, and eating disorders[19, 47–50].

Several interventions pioneered by experts in behavioral health and community health settings have successfully improved health outcomes in children exposed to adversity, including biomarkers associated with the stress response, as well as immune, metabolic, and epigenetic regulatory effects; targeting mechanisms of biological embedding of adversity may have implications for reducing subsequent development of diseases[51–55]. Many of these intervention programs have highlighted the beneficial role of a nurturant parent-child relationship[56]. Health care providers can play an instrumental role in promoting high quality parenting skills which can serve as a protective buffer during stressful childhood experiences[57]. Among adults with a history of childhood adversity, research shows that greater social support and reduced behavioral risk factors (i.e. drinking and smoking) can play an important role in enhancing the health and well-being of survivors[58]. Mental health treatment also has been effective[59].

### Limitations

Although this study helps to quantify the long-term impact of childhood exposure to ACEs in California, DALYs account for more than 90% of total estimated costs. Therefore, our cost estimate is quite sensitive to, and closely tracks the cost per DALY. Popular approaches to valuing DALYs are to survey how much a person would be willing to pay to avoid loss of a year of healthy life [e.g.,^60^, ^61^] or simply to divide the value of statistical life by the number of years of life that would be lost if the average person in the life valuation studies died (discounted to present value) [e.g.,^31^, ^62^, ^63^]. Hirth et al.[64] reviewed 42 studies on DALY/QALY values and found a wide range depending on methodology. Our use of $235,855 per DALY is in line with that study’s “intermediate median value” of $249,872 per DALY (inflated to 2017 dollars using the Employment Cost Index) from surveys, and lower than the $350,000 per DALY implied by a more recent meta-analytic study on the value of a statistical life[65]. At the same time, our use of a uniform value per DALY ignores evidence from disease- or country-specific studies which suggest the value per DALY is situational. For example, an intermediate value per DALY in surveys of asthma patients is $31,890 (in 2017 dollars), much lower than that for dialysis patients[66] and lower than the value used in our study. Given the uncertainty surrounding valuation of DALYs, we present the values separately from health expenditures and also present non-monetized estimates of the DALYs associated with ACEs.

Another source of variation in ACEs costs derives from our estimates of the ACEs-associated fractions (AAF), which describe associations but not necessarily causation. Our estimates are specific to California and may not carry over to other localities with different policies toward child abuse and mental health. Prior estimates of ACE associations with health outcomes across 13 BRFSS states[13, 24] yielded larger estimates of ACEs-associated fractions in those other states relative to California for obesity, asthma, and depression, but smaller estimates for heavy drinking and CVD (results available upon request). However, applying the 13-state estimates to California leaves the cost estimate relatively unchanged–ACEs exposure is associated with an estimated $9.7 billion in healthcare expenses and 378,000 DALYs valued at $89 billion.

This study relies on prior estimates of ACEs association with disease, healthcare expenses, and DALYs which have their own underlying limitations. The ACEs-associated fraction estimates are based on our prior study[13, 24] using retrospective ACEs data which are self-reported and subject to recall bias[67, 68]. Total associations with 4+ ACEs in our prior study are generally consistent with prior BRFSS studies for asthma and depression (e.g. [21][23]) and with results from a meta-analysis by Hughes et al.[69]; however, the odds of cardiovascular disease and COPD for 4+ ACEs are smaller in our study than in the seminal ACEs study which focused on a sample of mostly middle-class white adults in California [e.g., 18]. To the degree that ACEs exposure is under- or over-reported and because our analysis was unable to control for childhood background or the timing and intensity of ACEs, our estimates may misstate ACEs associations with outcomes and have a limited causal interpretation. Self-reports of health conditions in BRFSS may also be subject to recall bias and the underreporting of socially undesirable information. Bowlin et al.[70] report that the BRFSS may understate the prevalence of cardiovascular disease risk factors such as obesity and smoking. At the same time, this and other studies have found moderate sensitivity in BRFSS self-reports of diabetes and arthritis[71, 72] and BRFSS prevalence has been treated as a benchmark to test the possibility of using data on filled heart disease prescriptions as a proxy for prevalence[73]. However, to the extent that we underestimate the prevalence of the health conditions and risk factors, we may underestimate the AAF and costs of ACEs-associated health outcomes. For health costs, we relied on published healthcare expenses from Dieleman et al.[12] who used incomplete survey data requiring statistical modeling and scaling up to reflect national estimates and compensate for the lack of a census of US healthcare expenditures. The DALYs estimates share the limitations of the GBD estimates underlying them, notably the use of disability weights that are not tailored to high-income countries, the garbage codes in 15% of US cause-of-death records, and the use of models rather than California data sets to decompose nonfatal estimates by state. Finally, while our study estimates ACEs-related costs for many of the leading causes of death in the US, we did not examine a comprehensive list of all possible health outcomes. Thus, our estimates of health burden and costs may understate the true impact of ACEs exposure.

Despite these limitations, this study provides the first look at the adult health burden and healthcare costs to Californians associated with exposure to ACEs. Our estimates can be used to inform efforts to reduce the impact of ACEs and toxic stress in California. Future research could extend these results to other states or provide nationwide estimates.

## Supporting information

S1 Table Predicted change in probabilities of selected outcomes associated with ACEs exposure in California. (DOCX)

S2 Table Estimated personal healthcare spending and DALYs for selected conditions in CA, 2013. (DOCX)
