# Supplementary figures and images for: Adult health burden and costs in California during 2013 associated with prior adverse childhood experiences
Source: PLoS One. 2020 Jan 28;15(1):e0228019. doi: 10.1371/journal.pone.0228019 (PMC6986705; doi:10.1371/journal.pone.0228019)

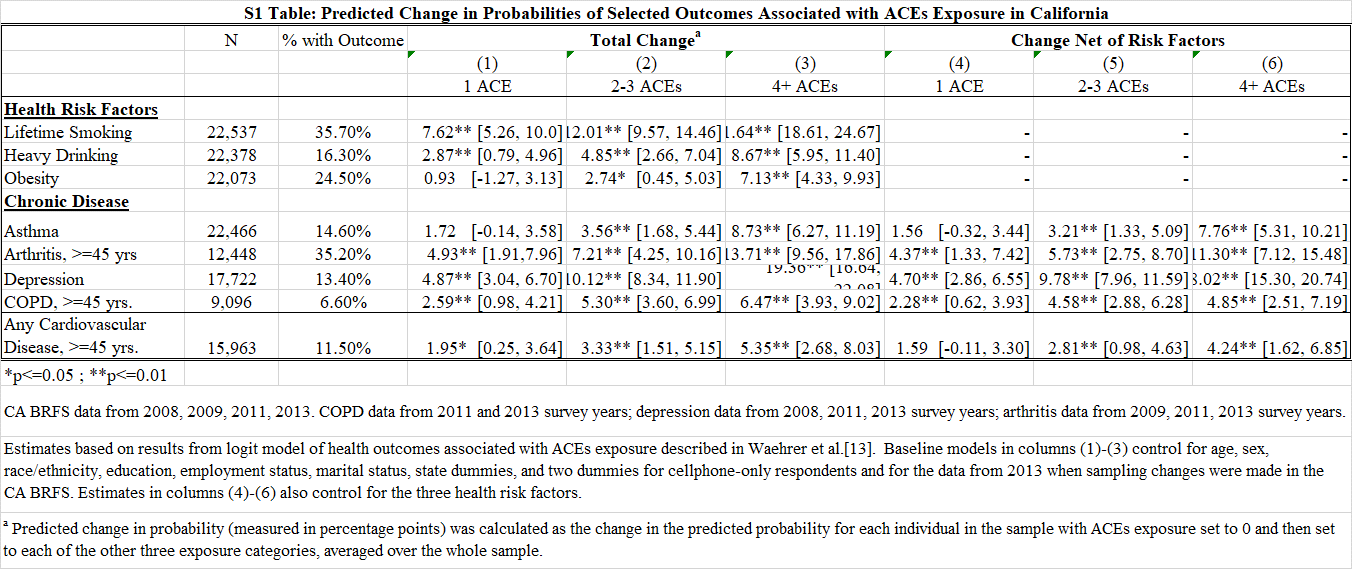

Supplement: S1 Table — (DOCX) [file pone.0228019.s001.docx]

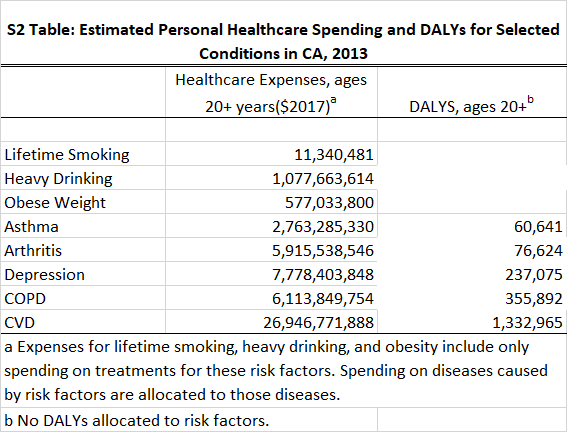

Supplement: S2 Table — (DOCX) [file pone.0228019.s002.docx]
